# Anisotropy of magnetic damping in Ta/CoFeB/MgO heterostructures

**DOI:** 10.1038/s41598-023-35739-8

**Published:** 2023-05-26

**Authors:** Bivas Rana, YoshiChika Otani

**Affiliations:** 1 Institute of Spintronics and Quantum Information, Faculty of Physics, Adam Mickiewicz University in Poznań, Uniwersytetu Poznanskiego 2, 61-614 Poznan, Poland; 2 Center for Emergent Matter Science, RIKEN, 2-1 Hirosawa, Wako, 351-0198 Japan; 3 Institute for Solid State Physics, University of Tokyo, Kashiwa, Chiba 277-8581 Japan

**Keywords:** Spintronics, Magnetic properties and materials

## Abstract

Magnetic damping controls the performance and operational speed of many spintronics devices. Being a tensor quantity, the damping in magnetic thin films often shows anisotropic behavior with the magnetization orientation. Here, we have studied the anisotropy of damping in Ta/CoFeB/MgO heterostructures, deposited on thermally oxidized Si substrates, as a function of the orientation of magnetization. By performing ferromagnetic resonance (FMR) measurements based on spin pumping and inverse spin Hall effect (ISHE), we extract the damping parameter in those films and find that the anisotropy of damping contains four-fold and two-fold anisotropy terms. We infer that four-fold anisotropy originates from two-magnon scattering (TMS). By studying reference Ta/CoFeB/MgO films, deposited on LiNbO_3_ substrates, we find that the two-fold anisotropy is correlated with in-plane magnetic anisotropy (IMA) of the films, suggesting its origin as the anisotropy in bulk spin–orbit coupling (SOC) of CoFeB film. We conclude that when IMA is very small, it’s correlation with two-fold anisotropy cannot be experimentally identified. However, as IMA increases, it starts to show a correlation with two-fold anisotropy in damping. These results will be beneficial for designing future spintronics devices.

## Introduction

Magnetic damping, one of the critical parameters of magnetic materials, governs the performance and operational speed of many proposed spintronics devices. It decides how fast the energy of the spin system sustaining the precessional magnetization motion dissipates into the nonmagnetic systems such as lattice, substrate, the environment through the interactions like magnon-magnon, magnon-electron, magnon-phonon and so on^1^. Initially, damping was thought to be a scalar quantity, i.e., isotropic in a magnetic material. Later, many theoretical studies^2–4^ predicted and experimental studies^5–9^ demonstrated that the damping is a tensor quantity and anisotropic. This essentially means that the damping depends on the magnetization orientation (known as *orientational anisotropy*). The anisotropy of damping becomes more prominent in magnetic materials with higher spin–orbit coupling (SOC) strength, which may originate from bulk and/or interfaces of magnetic thin film heterostructures.

Damping in magnetic thin films has many intrinsic and extrinsic sources. Using theoretical models^10–13^ and experimental reports^14,15^, the intrinsic component of damping *α*_int_ can be written as ${\alpha }_{int}=n\left({E}_{F}\right){\delta }^{2}{\tau }^{-1}$, where $n\left({E}_{F}\right)$ is the density of states (DOS) at the Fermi level, $\delta$ is the SOC strength and $\tau$ is the electron scattering time. It turns out that the intrinsic damping of a magnetic material can be optimized by adjusting these three parameters, i.e., by engineering electronic band structure at the Fermi level, as demonstrated by Schoen et al*.*^16^. The damping can show anisotropic behavior in the presence of anisotropic DOS at the Fermi level, as the shape of the Fermi surface depends upon the direction of magnetization because of SOC^8^. Likewise, anisotropic SOC strength may also generate anisotropy in damping^9^. However, the presence of sufficiently high scattering rates often smear the anisotropic damping behavior in many magnetic systems. Other intrinsic source of damping, such as magnon-phonon coupling^17^ (primarily observed in magnetostrictive films like Ni) do not impose anisotropy. In magnetic thin film heterostructures, the spin pumping (SP)^18–21^ into adjacent heavy metals with high SOC strength and spin memory loss (SML)^22,23^ at the interfaces are identified as additional sources of damping. However, both sources do not impose any anisotropy in damping. Apart from the above-mentioned intrinsic sources, there are several extrinsic sources of damping, such as Eddy current^24,25^ (observed in relatively thicker magnetic films) and two-magnon scattering (TMS). The Eddy current damping is not responsible for anisotropic damping behavior. TMS is observed because the uniform magnons (wavevector *k* ~ 0) are scattered from inhomogeneities or imperfections present at the interfaces and converted into degenerate nonuniform magnons (*k* ≠ 0)^26–28^. The TMS, observed for in-plane magnetization orientation, induces strong anisotropy in the damping, especially for ultrathin magnetic films, where interfacial effects become prominent^26,27,29^. Recently, Zhu et al*.* showed that the TMS in heavy-metal (HM)/ferromagnet/oxide heterostructures arises primarily at the HM/ferromagnet interface. In contrast, TMS at the ferromagnet/oxide interface is relatively weak. The TMS increases with the SOC strength and magnetic inhomogeneities at HM/ferromagnet interface^28^. Overall, the anisotropy in damping arises due to magnetization orientation-dependent anisotropy in DOS and SOC, and the anisotropic nature of TMS. Intrinsic damping is known as viscous damping, which means the resonance linewidth increases linearly with the resonance frequency. TMS, on the other hand, shows a complex nonlinear dependence on resonance frequency^30–32^. However, TMS was also found to show a linear dependence on frequency^30,33,34^. It is especially observed when the resonance linewidth is measured for a short range of frequency, or the low frequency phenomena (e.g. inhomogeneous line broadening) overshadow the nonlinear behavior, and/or contribution from TMS is relatively weaker than others. This makes it quite challenging to isolate intrinsic damping from the damping caused by TMS.

Recently the studies of various physical phenomena originated at the surfaces/interfaces of magnetic thin films^35–40^ owing to interfacial SOC are in high demand because of their possible potential applications in future spintronics devices. Naturally, investigation of the damping constants of magnetic thin films and their heterostructures, where interfaces play a key role on the damping constant, has also gained momentum in recent years. Efforts have been made to control the damping constant by thickness variation^41–43^, material engineering^16^, electric field^44,45^. Consecutively, orientational anisotropy also shows a route towards the control of damping just by changing the orientation of the magnetic field without replacing the magnetic film, which is quite appealing from the application point of view. In the current study, we report the anisotropy of damping in Ta/CoFeB/MgO heterostructures deposited on thermally oxidized Si and LiNbO_3_ substrates. We find that the anisotropy of damping is composed of four-fold and two-fold anisotropy terms. We infer that the four-fold anisotropy originates from TMS. On the other hand, the two-fold anisotropy that correlates with the in-plane magnetic anisotropy (IMA) of the films, originates from bulk SOC of CoFeB film.

## Results

### Device structure and measurement principle

**Figure 1:**
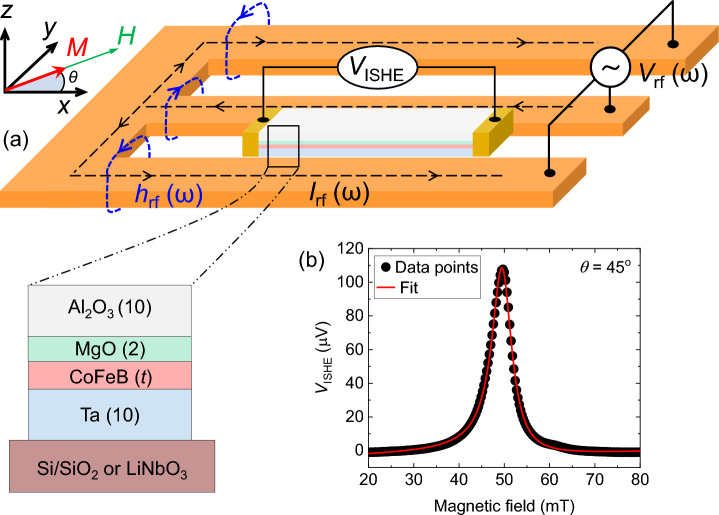
Device structure and measurement principle. (**a**) The schematic illustration represents the ferromagnetic resonance (FMR) measurement setup. A radio frequency current *I*_rf_ (ω) passes through a coplanar waveguide surrounding the rectangular-shaped multilayer film. The Oersted field *h*_rf_ (ω), induced by *I*_rf_ (ω), excites the FMR in CoFeB films at resonance condition given by Eq. (2). The magnetic field $H$ is applied in the film plane at an angle (*θ*) to the long axis of the film. The FMR signals are detected by measuring potential drop (*V*_ISHE_) across the rectangular multilayer film. Inset shows the cross-section of the multilayer film. (**b**) A typical ISHE signal measured from Si/SiO_2_/Ta/CoFeB(2.2)/MgO film at 5 GHz microwave frequency and 18 dBm (i.e., 63 mW) microwave power is presented. The solid line represents the fit to Eq. (1).

The sample fabrication and measurement principle are discussed in detail in the “Methods” section and can be found in references^45,46^. The devices for this study were fabricated from the multilayer stacks Ta(10)/Co_20_Fe_60_B_20_ (*t* = 1.6, 1.8, 2.0, 2.2)/MgO(2)/Al_2_O_3_(10) deposited on thermally oxidized Si[001] substrates, and Ta(10)/Co_20_Fe_60_B_20_ (*t* = 2.0)/MgO(2)/Al_2_O_3_(10) deposited on Y-cut LiNbO_3_ substrates^47^. The numbers in the parentheses indicate the thicknesses of the corresponding layers. For simplicity, the films are denoted as substrate/Ta/CoFeB(*t*)/MgO in this article. As described in the “Methods” section, a microwave current (*I*_rf_) from a signal generator, passing through the antenna, excites FMR in rectangular-shaped CoFeB films (Fig. 1a). The resonance signals are detected by measuring potential drop across the rectangular devices originated from SP and inverse spin Hall effect (ISHE).

Figure 1b represents a measured FMR signal (solid dots) from Si/SiO_2_/Ta/CoFeB(2.2)/MgO film. The microwave power for all the measurements were set well below the nonlinear regime (see Supplementary Fig. S1). The values of resonance field (*H*_0_) and resonance linewidth are determined by fitting the FMR signals to the following expression^48–50^:

1 $${V}_{\mathrm{ISHE}}={V}_{0}+\frac{{V}_{s}}{1+{\left(H-{H}_{0}\right)}^{2}/{\sigma }^{2}}+\frac{{V}_{a}\left(H-{H}_{0}\right)/\sigma }{1+{\left(H-{H}_{0}\right)}^{2}/{\sigma }^{2}}$$

Here, ${V}_{0}$ is the background of ${V}_{\mathrm{ISHE}}$, ${V}_{s}$ & ${V}_{a}$ are the weights of the symmetric Lorentzian and dispersive functions, respectively, and $\sigma$ is the half-width at half maximum (HWHM) of the FMR spectrum. The almost perfect symmetric Lorentzian shape of the ISHE signal ensures that the FMR is predominantly excited by the out-of-plane component of the microwave Oersted field^18^.

### Out-of-plane and in-plane magnetic anisotropies of the films

**Figure 2:**
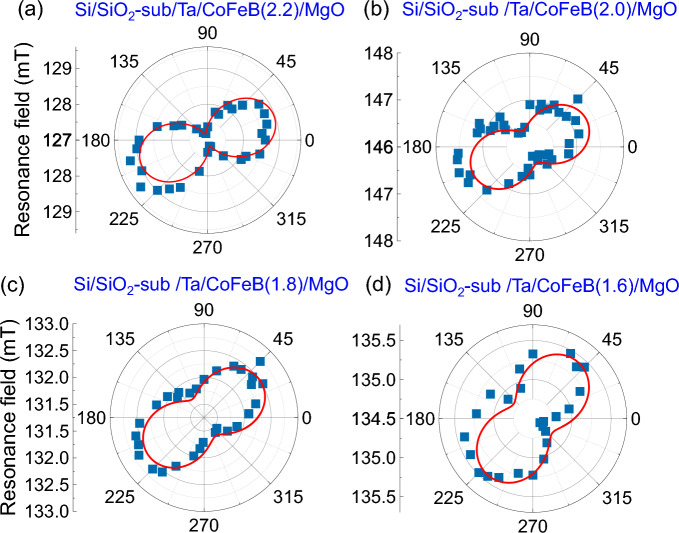
Resonance field versus in-plane magnetic field direction. The resonance field (*µ*_0_*H*_0_) versus in-plane magnetic field angle (*θ*) plot for Si/SiO_2_/Ta/CoFeB(*t*)/MgO films with CoFeB layer thickness *t* = 2.2 (**a**), 2.0 (**b**), 1.8 (**c**), and 1.6 nm (**d**). The resonance fields (*µ*_0_*H*_0_) were measured at microwave frequencies *f* = 8.5, 7.8, 6.5, 4.5 GHz and microwave powers of 18, 18, 16, 14 dBm for *t* = 2.2, 2.0, 1.8, and 1.6 nm, respectively. The solid curves are fits to the Eq. (2).

The out-of-plane and in-plane magnetic anisotropies of the studied films were characterized by measuring resonance signals for different orientations (*θ*) of the in-plane bias magnetic field, i.e., magnetization. In all the measurements the applied bias magnetic field was set much larger than the IMA field, which rules out the increment of FMR linewidth as a consequence of field dragging effect. The resonance field (*µ*_0_*H*_0_) corresponding to each FMR spectra is extracted by fitting with Eq. (1) and plotted as a function of *θ* (see Fig. 2). The *µ*_0_*H*_0_ versus *θ* data points are subsequently fitted to the following analytical equation^51^:

2 $${H}_{0}=-{H}_{k}+\frac{3}{2}{H}_{k}{\mathit{sin}}^{2}\left(\theta +\beta \right)-\frac{\left({M}_{s}-{H}_{p}\right)}{2}+\frac{1}{2}{\left[{H}_{k}^{2}{\mathit{sin}}^{4}\left(\theta +\beta \right)+({M}_{s}-{H}_{p}{)}^{2}+2\left({M}_{s}-{H}_{p}\right){H}_{k}{\mathit{sin}}^{2}\left(\theta +\beta \right)+4{\left(\frac{f}{{\mu }_{0}\gamma }\right)}^{2}\right]}^\frac{1}{2}$$

**Figure 3:**
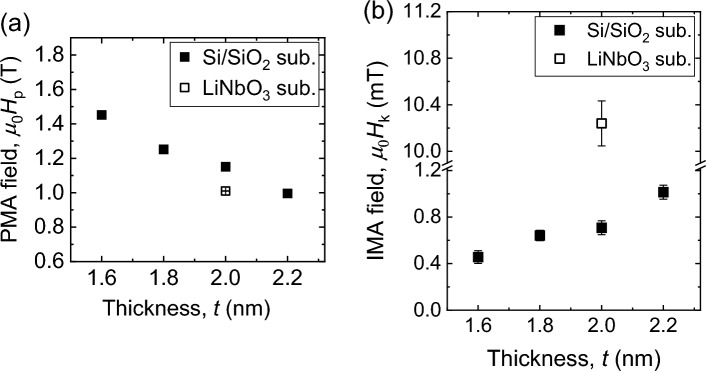
Anisotropy fields versus film thickness. The extracted values of PMA field (*µ*_0_*H*_p_) (**a**), and IMA field (*µ*_0_*H*_k_) (**b**) are plotted as a function of CoFeB film thickness. The error bars are included within the symbols.

Here, *γ* is the gyromagnetic ratio, *µ*_0_ is the permeability of free space, *f* is the microwave frequency, i.e., FMR frequency, *H*_k_ stands for the in-plane magnetic anisotropy (IMA) field with *β* being the direction of the IMA axis with respect to the long axes of the rectangular CoFeB structures, *M*_s_ is the saturation magnetization, and *H*_p_ stands for the perpendicular magnetic anisotropy (PMA) field. Figure 2 shows the polar plot of *µ*_0_*H*_0_ (solid points) as a function of *θ* for Si/SiO_2_/Ta/CoFeB(*t*)/MgO films. The angular variation of *µ*_0_*H*_0_ are well fitted with Eq. (2) as shown by the solid curves. It should be noted here that different microwave frequencies were used for different films so that the resonance fields for all the films fall in the range between 125 and 150 mT, close to the half of the maximum external magnetic field used for the measurements. This helps us to fit all the obtained FMR data very nicely with the Eq. (1). However, choosing different frequency for same sample should not affect the extracted values of the PMA and IMA fields. The extracted values of PMA fields *µ*_0_*H*_p_ are 0.996, 1.151, 1.252, 1.452 T, and IMA fields *µ*_0_*H*_k_ are 0.98 ± 0.06, 0.52 ± 0.06, 0.48 ± 0.04, 0.43 ± 0.05 mT for *t* = 2.2, 2.0, 1.8, 1.6 nm, respectively. Here saturation magnetization *µ*_0_*M*_s_ for all the films are considered to be 1.5 T^50^. Figure 3a shows the plot of *µ*_0_*H*_p_ as a function of *t*, which clarifies that PMA has purely interfacial origin as *µ*_0_*H*_p_ is inversely proportional to *t* (not shown). The IMA axes in the films with *t* = 2.2, 2.0, 1.8, and 1.6 nm, respectively, orient along 110°, 116°, 123°, and 148° to the long axes of the rectangular structures. It should be noted here that the extracted values of IMA fields (we say apparent IMA fields) are actually the resultant of the shape anisotropy of rectangular CoFeB films with dimension 200 × 12 µm^2^ and the actual IMA introduced by crystal structure. The shape anisotropy of all the rectangular CoFeB films are about 0.76 mT. By performing simple vector alzebra the actual IMA field values come out to be 1.01 ± 0.06, 0.71 ± 0.06, 0.64 ± 0.04, 0.46 ± 0.04 mT and IMA axes are oriented along 65°, 41°, 40°, and 62° to the long axes of the rectangular structures for *t* = 2.2, 2.0, 1.8, 1.6 nm, respectively. Figure 3b shows the plot of actual *µ*_0_*H*_k_ as a function of *t*. The *µ*_0_*H*_k_ increases with the increase of *t*, which means IMA may primarily have bulk origin. It is important to note here that the interfacial origin of IMA was reported in the previous works^51,52^. The strain developed inside CoFeB films during deposition and annealing could be one of the possible reasons behind IMA. However, more studies are required to unveil the origin. In our films, the IMA appears by annealing without an in-plane magnetic field, resulting in a different in-plane anisotropy axis in the films. Interestingly, the values of induced IMA are within 0.1% of the PMA.

### Anisotropy of damping

The total linewidth ($\sigma$) of a FMR spectra can be simply expressed as^16^:

3 $$\sigma ={\sigma }_{0}+\frac{2\pi \alpha }{\gamma }{f}_{\mathrm{FMR}}$$

Here, ${\sigma }_{0}$ is the frequency independent linewidth, originates from the inhomogeneous distribution of magnetic properties (such as PMA, IMA) of the ferromagnetic (FM) films, especially, in ultrathin films. The second term, which is proportional to the resonance frequency *f*, originates from the relaxation of spin angular momentum through: (1) intrinsic bulk SOC of FM film itself; (2) SP into the adjacent heavy metallic layer possessing high SOC strength; (3) interfacial SOC and interfacial SML. Hence to evaluate viscous damping $\alpha$, the extracted values of HWHM of FMR spectra are plotted as a function of *f* and fitted with a linear function. The values of $\alpha$ are then extracted from the slopes (Δ) of the linear fittings using the following expression^49^:

4 $$\alpha =\frac{\gamma }{2\pi }\Delta$$

**Figure 4:**
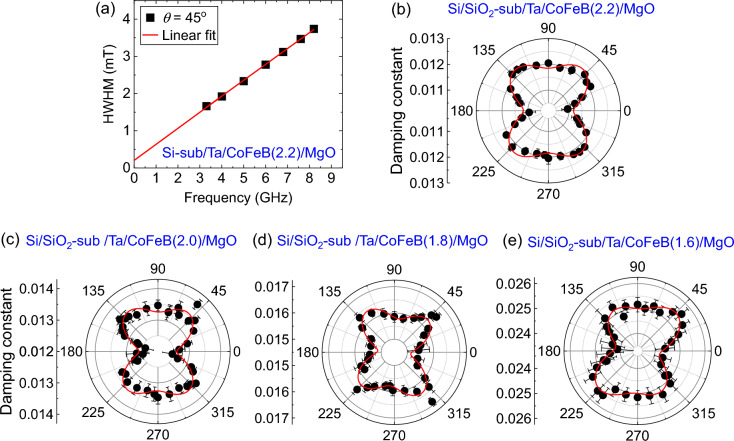
Angular variation of damping constant. (**a**) Resonance linewidth versus frequency plot for Si/SiO_2_/Ta/CoFeB(2.2)/MgO film at 45° magnetic field orientation. Solid line represents linear fit. The extracted values of the damping constant are plotted (filled points) as a function of the in-plane orientation of the magnetic field for Si/SiO_2_/Ta/CoFeB(*t*)/MgO films with *t* = 2.2 (**b**), 2.0 (**c**), 1.8 (**d**) and 1.6 nm (**e**). The solid curves represent the fits to Eq. (5).

Figure 4a represents the plot of HWHM as a function of *f* for Si/SiO_2_/Ta/CoFeB(2.2)/MgO film for 45° orientation of magnetic field, i.e., magnetization. Solid line represents linear fit. Please note that we have excluded here frequency dependent nonlinear term in the resonance linewidth [in Eq. (3)], which should be originated from TMS process. This is because all the linewidth versus frequency data show perfectly linear dependence. So, it is not really necessary to fit with nonlinear function. The reasons behind this linear behavior could be the short range of measurement frequency. To understand the angular dependent behavior of damping we plot the extracted values of $\alpha$ as a function of *θ* in Fig. 4b–e for Si/SiO_2_/Ta/CoFeB (*t* = 2.2, 2.0, 1.8, 1.6)/MgO films. The angular variation of damping shows a four-fold anisotropy overlapped with a two-fold anisotropy. Based on previous publications^6,8,26^ we assume that the observed angular behavior of *α* can be best fitted with the following phenomenological formula:

5 $$\alpha ={\alpha }_{0}+{\alpha }_{2}{sin}^{2}\left(\theta +{\beta }^{^{\prime}}\right)+{\alpha }_{4}{sin}^{2}2\left(\theta +{\beta }^{^{\prime}}\right) .$$

**Figure 5:**
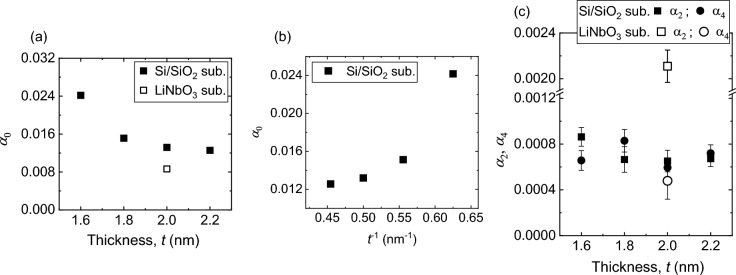
Damping coefficients versus film thickness. The extracted values of *α*_*0*_ for Ta/CoFeB/MgO films, deposited on Si/SiO_2_ and LiNbO_3_ substrates, are plotted as a function of (**a**) CoFeB film thickness *t* and (**b**) *t*^−1^. (**c**) Extracted values *α*_2_, *α*_4_ for all the Ta/CoFeB/MgO films are plotted as a function of *t*.

Here *α*_0_ is the *θ* independent isotropic component of damping; *α*_2_, *α*_4_ are the coefficients of two-fold and four-fold anisotropies, respectively; ${\beta }^{^{\prime}}$ is the offset angle. Figure 4b–e show that the angular variation of *α* is well fitted with Eq. (5). The extracted values of the coefficients of damping are plotted as a function of *t* in Fig. 5. The *α*_0_ increases monotonically with the decrease of *t* (see Fig. 5a). The SP and SML usually lead to inverse thickness (*t*^−1^) dependence of damping^22,53,54^, which is not observed in our case (see Fig. 5b). This indicates that the SP and SML are not solely responsible for the observed FM layer thickness-dependent damping behavior. So, TMS must have a significant contribution to the observed damping in our films, even though FMR linewidth shows linear dependence with frequency, according to the previous reports^30,34,55^. Although resonance linewidth produced by TMS should have a complex nonlinear dependence with frequency, these articles also mentioned that the linear frequency dependence of linewidth doesn’t guarantee the absence of the TMS process. It is especially true in the current study as the resonance linewidth is measured for a short range of frequency. Then it becomes quite difficult to completely isolate TMS contribution from viscous damping. Figure 5c shows that the coefficients *α*_2_, *α*_4_ vary randomly or, better to say, remain almost unchanged with *t* (solid points). The *α*_2_, *α*_4_ show their values up to 6% to *α*_0_, which is relatively low compared to the previous reports^6,8,9,56^. It turns out that our experimental method can probe and quantify even the presence of tiny anisotropy in the damping.

The anisotropic natures of TMS, SOC, and DOS can explain the anisotropic behavior of damping. The previous reports demonstrated that TMS could induce four-fold anisotropy in damping for cubic crystals^26,30,34^. In HM/ferromagnet/oxide heterostructures such as Ta/CoFeB/MgO, made of ultrathin FM layer, the significant contribution of TMS comes from the HM/ferromagnet interface due to the presence of strong interfacial SOC and interfacial roughness^28^. It is argued that the TMS contribution from ferromagnet/oxide interface is relatively weaker than the HM/ferromagnet interface. We infer that the extrinsic TMS at both (Ta/CoFeB and CoFeB/MgO) interfaces is responsible for the observed four-fold anisotropy of damping (*α*_4_) in our films. Usually, it can be expected that the lattice symmetry at the interface should replicate to the anisotropy in the damping caused by TMS. Now, all the layers in the studied films have cubic crystal structures [i.e. Ta, CoFeB, MgO have bcc, bcc and fcc crystal structures, respectively] after the annealing^57,58^. Therefore, it is quite natural that the TMS should have four-fold symmetry following the cubic symmetries at both interfaces. Another interesting point to note here that the TMS strength should show an inverse square relationship with *t*_CoFeB_ as discussed in the references^31,59,60^. In our case the measured range of microwave frequency is quite small and hence the linewidth versus frequency data cannot be fitted with the nonlinear function, responsible for TMS. As a result the nonlinear dependence of resonance linewidth with the frequency is overshadowed. Therefore, it is not possible for us to correctly evaluate the TMS strength. Although four-fold anisotropy in damping is solely originated from TMS, some part of the TMS induced damping may be leaked into the viscous damping (α_0_). That’s the reason α_0_ does not also show linear relationship with 1/*t*_CoFeB_ (see Fig. 5b). For the same reason α_4_, that primarily originates from TMS, does not show an inverse square relationship with *t*_CoFeB_, which is very clear from Fig. 5c and Supplementary Fig. S2.

Next we would like to find out the origin of two-fold anisotropy (*α*_2_) in damping. A correlation between *α*_2_ and IMA is expected to be observed if *α*_2_ originates from the anisotropies in the DOS and/or SOC in CoFeB films. However, *α*_2_ doesn’t show similar variation with *t* like IMA and the directions of *α*_2_ do not coincide with the axes of IMA (i.e. ${\beta }^{^{\prime}}\ne \beta$). In all the films, the directions of α_2 _ are along long axis of rectangular CoFeB film (i.e. $\beta^{^{\prime}} =0$). Therefore we cannot claim here that the anisotropies in the DOS and/or SOC in CoFeB films and its interfaces are responsible for the observed two-fold anisotropy. Hence, we studied a reference sample to investigate the origin of *α*_2_.

### Measurement of reference samples

**Figure 6:**
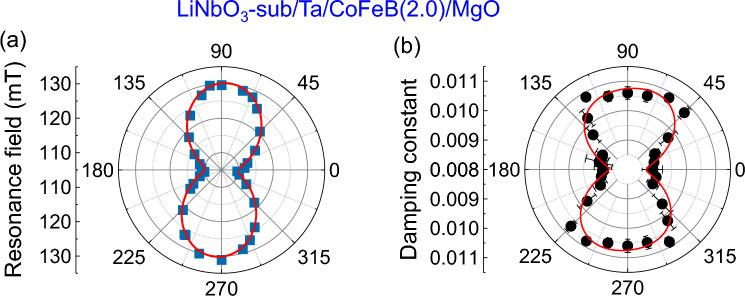
Angular dependence of resonance field and damping. (**a**) The resonance field (*µ*_0_*H*_0_) versus in-plane magnetic field angle (*θ*) plot for LiNbO_3_/Ta/CoFeB(2)/MgO film. The resonance fields (*µ*_0_*H*_0_) were measured at 8 GHz microwave frequency and 18 dBm microwave power. The solid curve is the fit to Eq. (2). (**b**) The extracted values of damping constant are plotted (filled points) as a function of in-plane orientation (*θ*) of magnetic field for LiNbO_3_/Ta/CoFeB(2)/MgO film. The solid curve is the fit to Eq. (5).

We measured the angular-dependent behavior of damping in a reference sample: Ta/CoFeB(2)/MgO deposited on Y-cut LiNbO_3_. By fitting the resonance field *µ*_0_*H*_0_ versus magnetic field angle *θ* to Eq. (2) (see Fig. 6a), we find that the PMA and IMA fields for this film are 1.01 T and 11 ± 0.19 mT, respectively (see Fig. 3a,b). The actual IMA field is 10.24 ± 0.19 mT and IMA appears along the *x*-axis. We observe a 15-fold increment in the IMA when the same film is deposited on the LiNbO_3_ substrate compared to Si/SiO_2_ substrate. Here, the LiNbO_3_ substrate promotes the crystallization axes of the CoFeB film so that the IMA is induced along the *x*-axis, i.e., along the long axis of the rectangular film. A slight reduction in PMA (which has interfacial origin) indicates that the IMA may primarily have bulk origin in the deposited films on LiNbO_3_ substrate; otherwise, IMA should have decreased like PMA. As plotted in Fig. 6b, the angular-dependent damping behavior shows the dominating two-fold orientational anisotropy along the IMA axis. The value *α*_2_ for LiNbO_3_/Ta/CoFeB(2)/MgO film, extracted from the fitting to Eq. (5), shows a three-fold increment compared to the *α*_2_ for Si/SiO_2_/Ta/CoFeB(2)/MgO film, suggesting a direct correlation between *α*_2_ and IMA. Notably, the direction of *α*_2_ is merged with the axis of IMA. Therefore, the anisotropy of SOC in CoFeB films is responsible for the observed two-fold anisotropy. Please note that IMA doesn’t cause the anisotropy in damping. However, as both of them originate from the anisotropy of SOC in CoFeB films, a correlation between these parameters is observed. The reduction of *α*_4_ for LiNbO_3_/Ta/CoFeB(2)/MgO film compared to the *α*_4_ for Si/SiO_2_/Ta/CoFeB(2)/MgO films discards any correlation between *α*_4_ and IMA, indicating its origin as TMS. Based on our observation, we argue that when IMA is very small (for Si/SiO_2_/Ta/CoFeB(*t*)/MgO films), it’s correlation with *α*_2_ cannot be experimentally identified. However, as IMA increases (for LiNbO_3_/Ta/CoFeB(2)/MgO films), it starts to show a correlation with *α*_2_ as both originate from bulk SOC of CoFeB films.

Previous study showed that the four-fold TMS can also be originated from the crystallographic defects^61^. In that case a correlation between four-fold TMS and four-fold magnetic anisotropy should be observed. However, this is unlikely in the present study as the films do not posses four-fold magnetic anisotropy. Another study showed that the two-fold anisotropy in damping can be originated from TMS, caused by the scattering from the artificial crystal defects created by oblique incidence of target material during film deposition^62^. We also exclude this mechanism as a correlation between two-fold anisotropy in damping and IMA is observed. Moreover, our films were deposited by sputtering method.

## Conclusions

In this work, we have investigated in-plane magnetization orientation-dependent anisotropy, i.e., orientational anisotropy of damping in Ta/CoFeB/MgO heterostructures deposited on thermally oxidized Si substrates. The damping constants are extracted by performing a ferromagnetic resonance (FMR) experiment excited by microwave current-induced Oersted field and detected through spin pumping (SP) and inverse spin Hall effect (ISHE) technique. The CoFeB films possess in-plane magnetic anisotropy (IMA), which increases with the film thickness suggesting its primary origin from the bulk spin–orbit coupling (SOC) of CoFeB films. The magnetization orientation-dependent damping consists of four-fold and two-fold anisotropies. We infer that the four-fold anisotropy originates from two-magnon scattering (TMS), which occurs because of the scattering of uniform magnons from inhomogeneities or imperfections at Ta/CoFeB and CoFeB/MgO interfaces to create degenerate nonuniform magnons. We do not observe any correlation between two-fold orientational anisotropy and IMA for Si/SiO_2_/Ta/CoFeB/MgO films, most probably because of the small value of IMA (i.e. small anisotropy in SOC strength). However, we find a direct correlation between two-fold orientational anisotropy and IMA in reference LiNbO_3_/Ta/CoFeB/MgO films, that contain strong IMA. This suggests that two-fold anisotropy in damping originates from SOC of CoFeB film. However, when IMA becomes relatively small in Si/SiO_2_/Ta/CoFeB/MgO films, its correlation with two-fold anisotropy is not experimentally observed. We believe that our work will help to design the orientational anisotropy in damping in future spintronics devices by engineering bulk and interfacial SOC strengths.

## Methods

### Sample fabrication

RF sputtering was used to deposit multilayer films Ta(10)/Co_20_Fe_60_B_20_ (*t* = 1.6, 1.8, 2.0, 2.2)/MgO(2)/Al_2_O_3_(10) on Si/SiO_2_(700) substrates and Ta(10)/Co_20_Fe_60_B_20_(2.0)/MgO(2)/Al_2_O_3_(10) on Y-cut LiNbO_3_ substrates at room temperature followed by vacuum annealing for 60 min at 280 °C temperature under 600 mT magnetic field applied along out-of-plane direction to the films. In the first step of fabrication, rectangular structures with lateral dimensions of 200 × 12 µm^2^ were defined on the deposited films with the help of maskless UV lithography followed by Ar^+^ ion milling down to the substrate. End point mass detector was used during the ion milling to ensure the right time to stop ion milling. In the second step, metal gate contacts at the edges of rectangular structures for measuring inverse spin Hall (ISHE) signals were prepared with the help of UV lithography and followed by the deposition of Ti(5)/Au(100) layer by electron beam evaporation. In the following step, 180-nm-thick Al_2_O_3_ layer was deposited by RF magnetron sputtering everywhere except on top of the edges of metal contacts made for measuring ISHE signals. In the final step, the microwave antennae for the excitation of FMR were prepared by maskless UV lithography followed by the deposition of Ti(5)/Au(200) layer by electron beam evaporation. It should be noted here that the microwave antennae are electrically isolated from rectangular CoFeB strips by 180-nm-thick Al_2_O_3_ layer.

### Experimental measurement

The FMR in CoFeB layers were excited by applying microwave current (i.e. *I*_rf_ (ω) from a signal generator) through the micrometer-sized antenna surrounding the rectangular shaped (200 × 12 µm^2^) magnetic film. This RF current through the antenna generates a microwave magnetic field (*h*_rf_) perpendicular to the film plane. The *H* is swept from − 320 mT to + 320 mT while keeping the frequency of RF current as fixed. At the resonance condition, a significantly large pure spin current (*I*_s_) is pumped from CoFeB layer into the adjacent Ta buffer layer, where *I*_s_ is converted into a transverse charge current (*I*_c_) through inverse spin Hall effect (ISHE) of Ta. The ISHE signal (*V*_ISHE_) is then measured by a nanovoltmeter. To study angular dependent behavior of damping the measurements were repeated for various in-plane orientations of bias magnetic field at a step of 7.5° or 15° with respect to the long axis of the rectangular devices. In all the measurements the applied bias magnetic field was much larger than the IMA field, which rules out the increment of FMR linewidth as a consequence of field dragging effect.

## Supplementary Information

Supplementary Figures.

## Data Availability

The datasets used and/or analysed during the current study available from the corresponding author on reasonable request.
