# Supplementary material for: Anisotropy of magnetic damping in Ta/CoFeB/MgO heterostructures
Source: Sci Rep. 2023 May 26;13:8532. doi: 10.1038/s41598-023-35739-8 (PMC10220008; doi:10.1038/s41598-023-35739-8)
Supplement: Supplementary file 1 — Supplementary Figures. [file 41598_2023_35739_MOESM1_ESM.docx]

Supplementary materials

**Anisotropy of magnetic damping in Ta/CoFeB/MgO heterostructures**

Bivas Rana^1,2*^, YoshiChika Otani^2,3^

^1^Institute of Spintronics and Quantum Information, Faculty of Physics, Adam Mickiewicz University in Poznań, Uniwersytetu Poznanskiego 2, Poznań 61-614, Poland

^2^Center for Emergent Matter Science, RIKEN, 2-1 Hirosawa, Wako 351-0198, Japan

^3^Institute for Solid State Physics, University of Tokyo, Kashiwa, Chiba 277-8581, Japan

^*^Correspondence should be addressed to: [bivran@amu.edu.pl](mailto:bivran@amu.edu.pl), bivasranaiitd@gmail.com

**S1. Resonance signals at different microwave powers**

To prove that the ferromagnetic resonances (FMR) are excited in linear regime, the FMR signals from Si/SiO_2_/Ta/CoFeB(2.2 & 2.0)/MgO films were measured as a function of microwave power. The resonance line shapes remain unchanged with the variation of microwave power. The plot of FMR signal amplitude as a function of microwave power shows that the FMR signals were measured in the linear regime for all the measurements presented in the manuscript. For instance, 18 dBm (i.e. 63 mW) microwave power was used for the angular dependent measurement of damping in Si/SiO_2_/Ta/CoFeB(2.0)/MgO film (marked as dotted line in Fig. S1(c)), which falls in the linear regime.

**Figure S1.** Measured FMR signals from Si/SiO_2_/Ta/CoFeB(*t*)/MgO films with *t* = 2.2 (a) and 2.0 nm (b) for various microwave power are plotted. (c) The ISHE signal amplitudes as a function of microwave power are plotted for Si/SiO_2_/Ta/CoFeB(2.0)/MgO film.

**S2. Four-fold anisotropy of damping versus inverse square thickness of CoFeB**

**Figure S2.** The four-fold anisotropies of damping are plotted as a function of the inverse square of CoFeB layer thickness in Si/SiO_2_/Ta/CoFeB(*t*)/MgO films.
